# GENETIC VARIANTS ASSOCIATED WITH BLOOD PRESSURE PHENOTYPES IN OLDER ADULTS

**DOI:** 10.1093/geroni/igad104.3369

**Published:** 2023-12-21

**Authors:** Lana Sargent, Dave Dixon, Marissa Mackiewicz, Elvin Price

**Affiliations:** Virginia Commonwealth University, Richmond, Virginia, United States; Virginia Commonwealth University, Richmond, Virginia, United States; Virginia Commonwealth University, Richmond, Virginia, United States; Virginia Commonwealth University, Richmond, Virginia, United States

## Abstract

African Americans (AAs) suffer from higher rates of uncontrolled HTN even when receiving commonly prescribed classes of antihypertensive medications. Variation in blood pressure (BP) in older adults is known to be associated with a variety of cardiovascular endpoints, yet our understanding of underlying mechanisms is limited. This study aimed to determine if HTN genetic variants influence blood pressure variation within a population of AA older adults taking antihypertensives. Community-dwelling older adults from the Translational Approaches to Personalized Health (TAPH) cohort were recruited to enroll in a 24-hour ambulatory BP monitoring (ABPM) phenotyping study. The Quant Studio 12-K Flex system was used for genotyping participants for three previously established CACNA1C and CACNB2 genetic risk alleles (rs758173, rs2228645, and rs2357790). 69 participants had ABPM data that passed the minimum 80% successful readings threshold: age=68□6, Female=50.3%, Black=73%, White=21%, and Other=6%. ABPM revealed 50.7% controlled HTN, 27.5% Sustained uncontrolled HTN, 14.5 % White coat uncontrolled HTN and 7.2% Masked uncontrolled HTN. ANOVA and Linear regression models found that the CACNB2 genetic risk allele rs2228645 broadly impacts ABPM Phenotypes (C/C SBP= 120.8+/-12.9 mmHg, C/T SBP= 125.73+/-13.5, and T/T SBP= 140+/-34.8 mmHg, p=0.011), mean awake 24-hour SBP (C/C SBP= 124+/-12.7 mmHg, C/T SBP= 133.5+/-14.8, and T/T SBP= 143.7+/-25.6 mmHg, p=0.012), and mean sleep SBP (C/C SBP= 114+/-16 mmHg, C/T SBP= 125.4+/-15, and T/T SBP= 136+/-50 mmHg, p=0.024). CACNA1C and CACNB2 genetic variants influence blood pressure phenotypes in older AAs. Determining genetic variants that affect antihypertensive effectiveness could lead to improved antihypertensive therapy for individuals with HTN/CVD.

